# Outperforming piezoelectric ultrasonics with high-reliability single-membrane CMUT array elements

**DOI:** 10.1038/s41378-022-00392-0

**Published:** 2022-06-02

**Authors:** Eric B. Dew, Afshin Kashani Ilkhechi, Mohammad Maadi, Nathaniel J. M. Haven, Roger J. Zemp

**Affiliations:** Department of Electrical and Computer Engineering, University of Alberta, Edmonton, Canada

**Keywords:** Electrical and electronic engineering, Sensors, Electronic devices

## Abstract

It has long been hypothesized that capacitive micromachined ultrasound transducers (CMUTs) could potentially outperform piezoelectric technologies. However, challenges with dielectric charging, operational hysteresis, and transmit sensitivity have stood as obstacles to these performance outcomes. In this paper, we introduce key architectural features to enable high-reliability CMUTs with enhanced performance. Typically, a CMUT element in an array is designed with an ensemble of smaller membranes oscillating together to transmit or detect ultrasound waves. However, this approach can lead to unreliable behavior and suboptimal transmit performance if these smaller membranes oscillate out of phase or collapse at different voltages. In this work, we designed CMUT array elements composed of a single long rectangular membrane, with the aim of improving the output pressure and electromechanical efficiency. We compare the performance of three different modifications of this architecture: traditional contiguous dielectric, isolated isolation post (IIP), and insulated electrode-post (EP) CMUTs. EPs were designed to improve performance while also imparting robustness to charging and minimization of hysteresis. To fabricate these devices, a wafer-bonding process was developed with near-100% bonding yield. EP CMUT elements achieved electromechanical efficiency values as high as 0.95, higher than values reported with either piezoelectric transducers or previous CMUT architectures. Moreover, all investigated CMUT architectures exhibited transmit efficiency 2–3 times greater than published CMUT or piezoelectric transducer elements in the 1.5–2.0 MHz range. The EP and IIP CMUTs demonstrated considerable charging robustness, demonstrating minimal charging over 500,000 collapse-snap-back actuation cycles while also mitigating hysteresis. Our proposed approach offers significant promise for future ultrasonic applications.

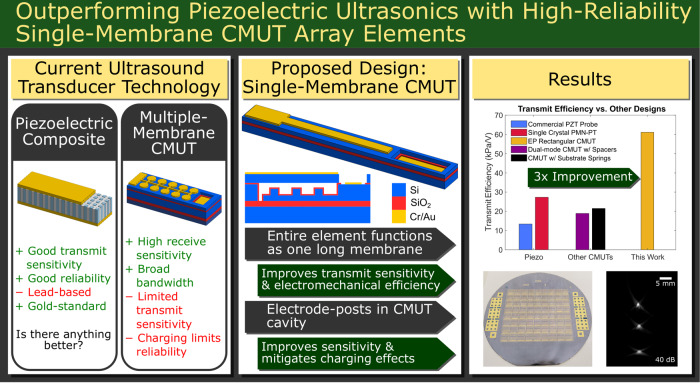

## Introduction

Capacitive micromachined ultrasound transducers (CMUTs) offer many potential advantages for next-generation ultrasound, including potential for integration with microelectronics, broad bandwidth, and outstanding receive sensitivity. Although CMUTs have been investigated for over 25 years^1^, widespread implementation in commercial ultrasound systems has been hindered by long-standing reliability and performance challenges associated with dielectric charging^2^ and operational hysteresis^3^. Despite much progress in the field, there are still unmet needs to improve long-term reliability, electromechanical efficiency, and performance for future ultrasound imaging applications.

**Fig. 1:**
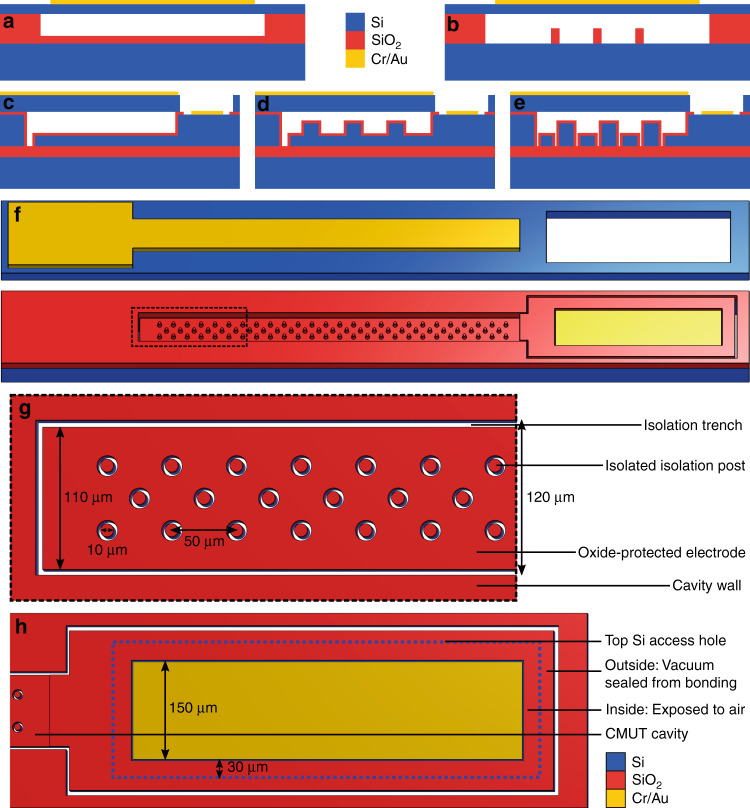
Single-membrane CMUT designs. **a** Cross-section of a typical contiguous dielectric (CD) CMUT cell. **b** Cross-section of the ‘PostCMUT’ architecture proposed by Huang et al^17^. **c** Cross-section of the fabricated CD rectangular CMUT. **d** Cross-section of the fabricated electrode-post (EP) rectangular CMUT. **e** Cross-section of the fabricated rectangular CMUT with a modified isolated isolation post (IIP) architecture including additional insulation. **f** Exploded view of an IIP rectangular CMUT model, showing important features on the bottom electrode. Note that the film thicknesses and membrane length are not to scale. **g** Close-up view of the cavity showing IIP configuration and relevant dimensions. **h** Close-up view of the bond pad region, with the blue outline representing the access hole in the top silicon. A vacuum seal is maintained for all areas outside of this region, including the CMUT cavity

In most CMUT designs, a dielectric layer is placed between the top and bottom electrodes to prevent short circuiting during operation, as illustrated in Fig. 1a. Dielectric charging occurs when charges are trapped on the surface of or within this layer due to the high electric fields associated with device operation. This effect can lead to altered device performance and even permanent failure. Hysteresis occurs in CMUTs when the applied voltage is increased beyond a threshold and the membrane collapses. Due to the increased electric field upon collapse, the bias voltage must be reduced to a ‘snap-back’ voltage to un-collapse the membrane. As a result, behavior between the ‘snap-down’ and snap-back voltage depends on the previous position of the membrane. The electromechanical efficiency of CMUTs in pre-collapse mode is highest just below the collapse voltage point. However, this is also a point of great instability, and operating near the collapse voltage can result in unpredictable behavior owing to hysteresis effects.

The current paradigm for designing CMUTs involves an ensemble of small membranes electrically connected to form an element within an array. These membranes oscillate to transmit or detect ultrasound signals. The dimensions of these individual membranes are selected to achieve the desired resonance frequency while maintaining an overall desired element size within an array. However, this approach has several drawbacks. Due to nonuniformities during fabrication, these membranes may collapse at different voltages^4^. To avoid unwanted hysteresis and further dielectric charging, the CMUT must be operated well below the mean collapse voltage, where electromechanical efficiency and immersion performance are poor^5^. Thus, although electromechanical coupling efficiencies (*k*_*T*_^2^) of up to 0.85 have been achieved very close to the collapse voltage, at more practical operating voltages (80–90% of collapse voltage), *k*_*T*_^2^ values of 0.3 or lower are typical^4–6^. These issues are compounded by suboptimal fill factor, particularly for sacrificial release fabrication processes^7^, and thus a low average displacement. Moreover, these small membranes can oscillate out of phase with each other owing to mutual acoustic coupling effects^8^ and Rayleigh-Bloch waves^9^. This can reduce overall transmit and receive sensitivity and create unwanted complex dynamics.

Collapse-mode operation has previously been proposed to improve the output and receive sensitivity of CMUTs^10,11^. However, collapse-mode operation is more susceptible to dielectric charging issues than pre-collapse operation since much larger electric fields are generated when in a collapsed state. Thus, reliability concerns from charging and hysteresis impact device performance by discouraging operation in higher efficiency regimes.

Much work has been done with the aim of understanding and mitigating dielectric charging effects^6,12,13^. This work has included research into film quality^14^, surface roughness, and deposition recipes to mitigate charging^15^. However, even with the best films, dielectric charging cannot be completely eliminated. Additional work has included architectural modifications. Huang et al.^16,17^ fabricated CMUT cells with an additional lithography step to pattern the insulating dielectric layer into small ‘isolation posts’ (Fig. 1b). Although these oxide posts were still susceptible to charging, they were small enough that the charges trapped in them had a minimal effect on the operation of the ‘PostCMUTs’. Additionally, the post height was designed to minimize hysteresis, as the membrane would contact the posts instead of collapsing fully. However, these devices had considerably lower transmit and receive sensitivity than conventional CMUTs without posts^17^. Additionally, as PostCMUTs do not have a contiguous dielectric insulating layer, this architecture is more susceptible to electrical shorting from any particles trapped within the gap, as described by Greenlay^18^.

Several groups have extended this idea, using dielectric posts or spacers to mitigate charging while patterning the top or bottom electrode to minimize the electric field through the post structures^18–20^. This approach further addresses charging by reducing the opportunities for charge to become trapped within the dielectric post, albeit at the expense of a reduced active area within each CMUT cell. This architectural modification has been incorporated into an ensemble of circular membranes by Mahmud et al.^19^ using anodic bonding with a borosilicate glass substrate, and hexagonal membranes by Machida et al.^20^ using a sacrificial release process.

Using the same principle, we previously incorporated ‘isolated isolation posts’ (IIPs) into circular CMUT cells using a fusion bonding process^18^. Similar to isolation posts, these IIPs prevented membrane collapse, thus precluding hysteresis and mitigating charging. Additionally, they were electrically isolated, minimizing the likelihood of the posts themselves charging. While this approach demonstrated minimal, if any charging, the overall electromechanical efficiency was impacted by membranes within an element collapsing at different voltages. We also found that some devices were prone to breakdown. This may have been due to unwanted particles in the gap or missing posts, along with an exposed bottom electrode where post structures were absent. Any instances of breakdown are problematic, as a single membrane failure within an element can lead to the malfunction of the whole element.

Other prior work has sought to improve CMUT performance by avoiding the multiple-membrane architecture. P. Zhang et al. investigated CMUT array elements composed of a single rectangular membrane^21^. These devices demonstrated promising results in air at diagnostic frequencies with relatively minor dielectric charging. However, neither charging nor hysteresis were completely eliminated, and electromechanical efficiency was not fully characterized. Other architectural modifications^22^ have been proposed to improve transmit output, including piston-like CMUTs with substrate-embedded springs^23^. Such devices can enable a single membrane per element, but long-term reliability was not explored.

Given that individual CMUT membranes can achieve electromechanical efficiency approaching unity near the collapse voltage, we hypothesized that we could achieve high electromechanical efficiency with CMUT architectures incorporating a single long rectangular membrane per array element. In our approach, the desired resonance frequency of an element is determined by the width (short axis dimension) and thickness of the membrane.

This work seeks to develop CMUT array elements that are both highly reliable and have excellent performance in immersion by combining the potential performance benefits from rectangular single-membrane CMUTs with architectural modifications to improve reliability. In particular, three single-membrane CMUT architectures are fabricated and compared in terms of reliability and performance.

The first architecture investigated is a contiguous dielectric (CD) rectangular CMUT, as illustrated in Fig. 1c. The second fabricated architecture is similar to the ‘PostCMUT’ design (Fig. 1b) demonstrated by Huang et al. However, this design features some important differences to improve performance. Our proposed ‘electrode-post’ (EP) architecture includes contiguous dielectric insulation throughout the cavity for protection of the bottom electrode. Additionally, the electrode-posts are patterned into the bottom silicon, as depicted in Fig. 1d. This is advantageous, as it leads to a reduced effective gap between electrodes in these regions and thus a greater electrostatic force applied to the membrane. The final rectangular CMUT architecture characterized involves isolated isolation posts (IIPs), similar to our previous work. As shown in Fig. 1e, this architecture is also modified with contiguous insulation.

Aside from device performance and reliability benefits, our work also includes a modified wafer-bonding fabrication process to improve fabrication yield. Our work demonstrates the achievement of difficult simultaneous requirements of high yield, high electromechanical efficiency, high acoustic output, sensitive receive operation, low hysteresis, and charging-free operation over long testing procedures. These results demonstrate the potential to outperform current piezoelectric technologies. This could be of considerable importance given the multibillion-dollar ultrasound transducer market.

## Materials and methods

### Device design

Each CMUT architecture was designed with a single rectangular membrane that was 3.0 mm long, 120 µm wide (1:25 aspect ratio), and 5 µm thick. The total height of the vacuum cavity was designed to be 500 nm, similar to some of our previous work^21^. In the cases of the electrode-post (Fig. 1d) and isolated isolation post architectures (Fig. 1e), the posts were designed to be 320 nm tall, with 180 nm of space for the membrane to deflect before contacting the posts. These dimensions were selected to allow the posts to ‘catch’ the deflecting membrane immediately before it collapsed, preventing hysteresis. A parallel-plate analytical model was used to predict the collapse point of these designs and provide insights into the effect of EPs and IIPs on device sensitivity (see Suppl. 1)^17,24–26^. Notably, this model predicts improved sensitivity of EP CMUTs compared to CD CMUTs, IIP CMUTs, or PostCMUTs. Finally, the oxide insulation layer in the cavity was 360 nm thick.

A diagram of a rectangular CMUT with IIPs is displayed in Fig. 1f. The posts present in our EP and IIP architectures were arranged as shown in Fig. 1g. Designs with denser configurations of posts were explored in IIP devices; however, this led to a reduced bottom electrode area and lower electromechanical efficiency without noticeable advantages.

Preserving the bottom electrode area was also a consideration when designing the width of the trenches surrounding each isolated isolation post. However, etching very thin trench diameters through relatively thick device layers can lead to challenges during lithography, etching, and cleaning steps, as observed in ref. ^18^. For this reason, most fabricated IIP devices were designed with 2–3 µm trenches.

Finally, a trench was etched into the bottom electrode to electrically isolate each CMUT element from the surrounding parasitic capacitance. To isolate the device from as much parasitic capacitance as possible without compromising the hermetic seal, the trench was etched inside the CMUT cavity and around the bond pad access holes, as shown in Fig. 1h.

### Process development

As the proposed single-membrane CMUT architectures have very long membranes, a wafer-bonding process is ideal for fabricating these devices. Fabrication of large membranes using a sacrificial release process is challenging due to large residual stresses and difficulties during membrane release, typically leading to stiction or broken membranes^27^. To fabricate IIP devices, patterning of the bottom electrode is required to electrically isolate the isolation post. Therefore, we used a wafer-bonding process with two silicon-on-insulator (SOI) wafers. In order to fabricate contiguous dielectric CMUTs, as well as our EP and IIP architectures on the same wafer, some differences were required from previously published double-SOI fusion bonding processes.

The original isolation post architecture demonstrated by Huang to mitigate charging consisted of small dielectric posts on an otherwise exposed bottom electrode. Our proposed EP architecture iterates on this design by adding further insulation to the bottom electrode (among other changes), reducing the likelihood of electrical shorting. However, as demonstrated by Christiansen et al.^28^, simply adding another thermal oxidation step leads to protrusions in the oxide. These oxide cusps lead to bond voids, considerably reducing bonding yield. Christiansen et al. proposed an oxide-oxide bonding process which improved bonding yield while insulating the CMUT cavity. However, using this process to fabricate architectures with a patterned bottom electrode requires aligned bonding. Aligned bonding is often associated with coarser alignment tolerances and is therefore not ideal for fabricating high-frequency devices or closely packed arrays.

Recently, Greenlay demonstrated a double-SOI wafer-bonding process for fabricating circular CMUTs with isolated isolation posts^18^. This process featured a single oxidation step, which was then patterned into the cavity and isolation post structures with two mask steps. As there was a single oxidation step, there were no oxide protrusions, and the bonding yield was good. However, this process left the bottom electrode exposed, and breakdown was a problem for these devices^18^.

In this work, we present a double-SOI wafer-bonding fabrication process that allows for the fabrication of fully insulated CD, EP, and IIP CMUT architectures with no oxide cusps and excellent bonding yield. This is achieved by patterning structures into the device layer of the bottom SOI wafer using plasma etching prior to a single oxidation step. A secondary benefit of performing etching steps prior to oxidation is that the oxide layer is not exposed to the high electric fields present during reactive ion etching (RIE) steps. As a result, the oxide avoids precharging during fabrication.

### Transducer fabrication

**Fig. 2:**
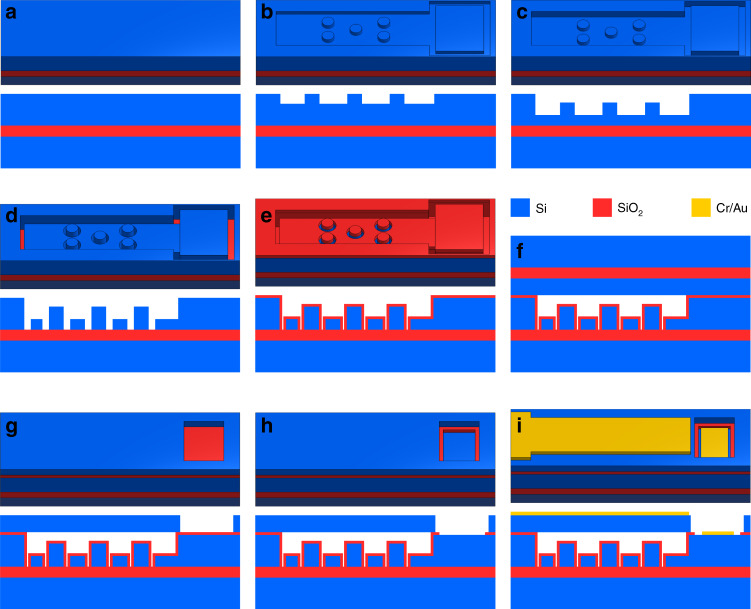
Overview of device fabrication process. Simplified 3D drawing (above) and cross-sectional view (below) of a rectangular IIP CMUT during each fabrication step. **a** Bottom SOI wafer before processing. **b** First lithography and etching, defining posts from the bottom electrode. **c** Second patterning step, etching down the entire cavity. **d** Third mask step and DRIE, etching through the device layer, isolating IIP structures, and completing trenches around elements. **e** Thermal oxidation of the bottom SOI wafer. **f** Fusion bonding and annealing of the top and bottom SOI wafers. **g** Handle removal and fourth lithography step, etching through the membrane to access the bottom electrode. **h** Fifth mask step, etching through thermal oxide to access the bottom electrode. **i** Contact metal deposition and patterning

The wafer-bonding process used to simultaneously fabricate IIP, EP, and CD rectangular CMUTs on the same wafer is summarized in Fig. 2. This process was performed using different 100 mm SOIs for the top and bottom wafers. Device layer specifications of the top SOI determine the membrane properties, while the bottom device layer determines the resistivity of the bottom electrode. The bottom substrate was purchased from Silicon Valley Microelectronics (Santa Clara, CA, USA), specified to have a 10 µm device layer, <0.005 Ωcm device resistivity (arsenic doped), 300 nm BOX thickness, and a 500 µm handle. Top SOI wafers were purchased from Ultrasil (Hayward, CA, USA), designed with a 5 µm thick device layer, 0.001–0.005 Ωcm device resistivity (boron doped), a 400 µm handle, and a 0.5 µm BOX layer. Additional details are provided on process steps in Suppl. 2^29–31^.

Processing begins on the bottom substrate (Fig. 2a) with standard piranha cleaning, followed by the first lithography. The first lithography step defines the post structures in EP and IIP CMUTs, as shown in Fig. 2b, while in contiguous dielectric elements, the entire cavity is left exposed. Exposed silicon is then etched by 320 nm using a Cl_2_-based reactive ion etching (RIE) recipe. The second mask step exposes the entire CMUT cavity (Fig. 2c), which is etched by 180 nm using Cl_2_-based RIE. This etches down post structures, allowing room for the membrane to deflect. The third lithography exposes trenches around IIPs while protecting the remainder of the cavity with photoresist. An additional trench is exposed in all architectures to isolate the bottom electrode from parasitic capacitance. Bosch deep reactive ion etching (DRIE) is used to etch these trenches through the device layer with a high aspect ratio (Fig. 2d).

Following DRIE, a 360 nm SiO_2_ layer is grown on the bottom substrate using dry oxidation, as illustrated in Fig. 2e. Thermal oxidation occurs at 1100 °C immediately after a standard piranha clean. The processed bottom wafer and top SOI wafer are prepared for fusion bonding with RCA cleaning (all steps). Immediately following the cleaning process, the wafers are fusion bonded in 5 mTorr vacuum (CB6L, SÜSS MicroTec, Garching, Germany), as depicted in Fig. 2f. The bonded wafer pair is then annealed at 1100 °C to form covalent bonds.

**Fig. 3:**
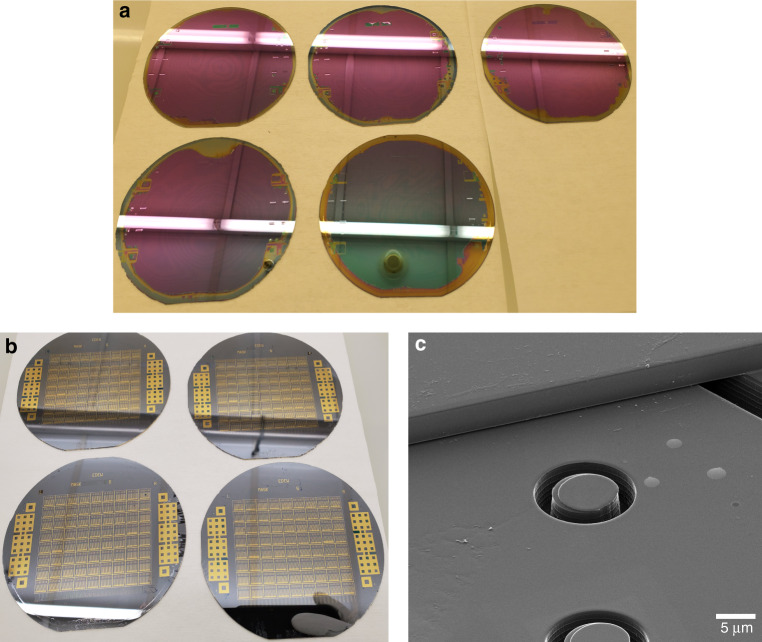
Fabricated devices. **a** Wafers photographed after handle removal with a visible BOX layer. None of the wafers pictured have any voids in the device area, illustrating consistent good bonding yield across many wafers. Note that the dark circle visible in the center of the bottom-right wafer is a reflection of the cleanroom ceiling and not a defect on the wafer. **b** Photograph of completed wafers. **c** Helium ion microscopy image of a rectangular IIP CMUT cavity showing isolated isolation posts (center), the CMUT membrane, and the isolation trench (right)

Before bulk etching of the top SOI handle to release the membrane, PECVD oxide is deposited on the bottom wafer handle for protection. Bulk etching of the top wafer handle is achieved using tetramethylammonium hydroxide (TMAH). After handle removal, only the buried oxide (BOX) layer and bonded device layer remain of the top SOI, making it easy to notice any bond voids. Fig. 3a displays a photograph of wafers prior to BOX layer removal, with no visible voids in the device area. The BOX layer is then removed using buffered oxide etch (BOE).

The fourth lithography (Fig. 2g) step defines access holes in the silicon membrane, which are etched using DRIE. Figure 2h illustrates the fifth patterning step, which etches access holes in the thermal oxide using RIE, (CHF_3_ and O_2_) exposing the bottom electrode. Metal contacts (Al or Cr/Au) are deposited to a thickness of 250 nm using magnetron sputtering. Finally, the contacts are patterned and wet etched (Fig. 2i). Completed wafers are shown in Fig. 3b. A helium ion image of a fabricated rectangular IIP CMUT cavity is given in Fig. 3c.

## Results

### Electrical characterization

**Fig. 4:**
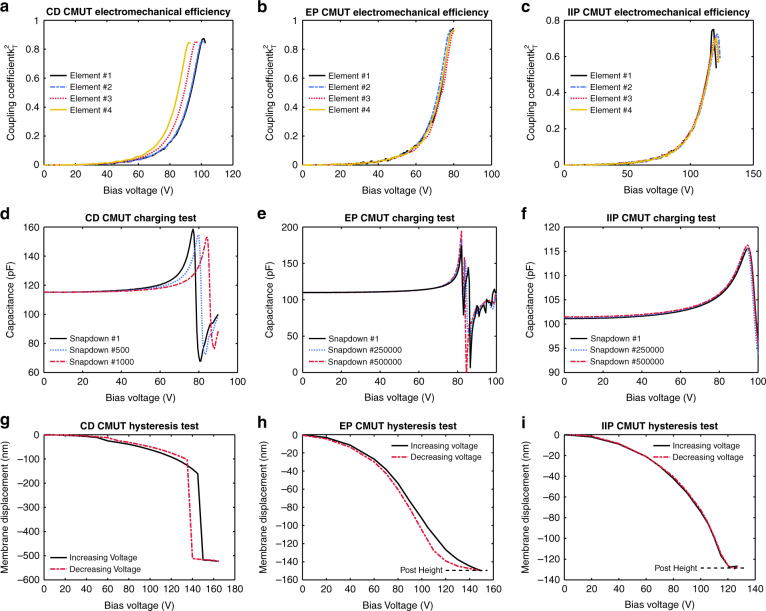
Performance and reliability testing. **a**–**c** Electromechanical efficiency as a function of bias voltage for each fabricated rectangular CMUT architecture as calculated from capacitance-voltage data. **d**–**f** Capacitance–voltage measurements before and after many consecutive snap-down events intended to charge each device. Shifts in the CV curve (as seen with the contiguous dielectric CMUT) are indicative of charging. **g**–**i** Static membrane deflection in each architecture as a function of bias voltage. Bias was first increased from zero to the maximum and then decreased from the maximum to zero

The performance of fabricated CMUTs was tested by measuring capacitance as a function of bias voltage. For each device architecture, the bias voltage was swept past the collapse voltage of the device, and the capacitance was measured using a 2.0 MHz AC signal with an amplitude of 30 mV. The measured capacitance–voltage (CV) curves were used to determine the electromechanical efficiency of the devices as outlined in^4,24^. An array of elements on a die was investigated for each rectangular CMUT architecture using this technique, and the CV curve of every element on the die was measured. Calculated from these measurements, the *k*_*T*_^2^ value as a function of bias voltage is plotted for example devices from the CD, EP, and IIP dies in Fig. 4a–c.

CD single-membrane CMUTs achieved a mean *k*_*T*_^2^ value near the collapse voltage of 0.84, with a standard deviation of 0.02. The best performing element on this die had a peak electromechanical efficiency of 0.87, while the best performing CD device on the wafer exhibited a peak electromechanical efficiency of 0.91. For IIP devices, the mean *k*_*T*_^2^ value close to the collapse voltage was 0.69, with a standard deviation of 0.03. The best performing IIP element demonstrated a peak electromechanical efficiency value of 0.75. Finally, rectangular EP CMUTs had a mean electromechanical efficiency of 0.92, close to the snap-down voltage, with a standard deviation of 0.02. The best performing device had an electromechanical efficiency of greater than 0.95, and all elements on the die achieved an electromechanical efficiency of better than 0.88.

CV testing was also used to characterize the long-term charging performance of the different architectures. This was accomplished by performing a large number of voltage sweeps beyond the collapse voltage of each device, inducing charging from the large electric fields. Fig. 4d–f displays the results of this charging test.

As expected, the CD devices were prone to charging, with noticeable shifts in the CV curve after 500 snap-down events. By contrast, both the EP and IIP CMUTs did not charge significantly even after 500,000 sweeps beyond the snap-down voltage and 18 h of exposure to high electric fields. Charging tests had good repeatability, with EP and IIP devices consistently demonstrating robustness to charging compared to CD CMUTs. These devices were all fabricated on the same wafer, so factors such as oxide quality were controlled to a large extent.

### Optical profilometry

Each CMUT design was investigated for hysteresis using an optical profilometer (NewView 5000, Zygo Corporation, Middlefield, CT) to measure membrane deflection. The CMUT element was actuated with a DC bias, which was increased monotonically from zero to beyond the collapse voltage and then decreased back to zero. Static membrane deflection was recorded at each bias voltage, with special attention to record the snap-back voltage when applicable. As demonstrated in Fig. 4g–i, the CD CMUTs exhibited hysteresis, the EP devices displayed minimal hysteresis, and the IIP devices were free from hysteresis.

### Laser Doppler vibrometry

**Fig. 5:**
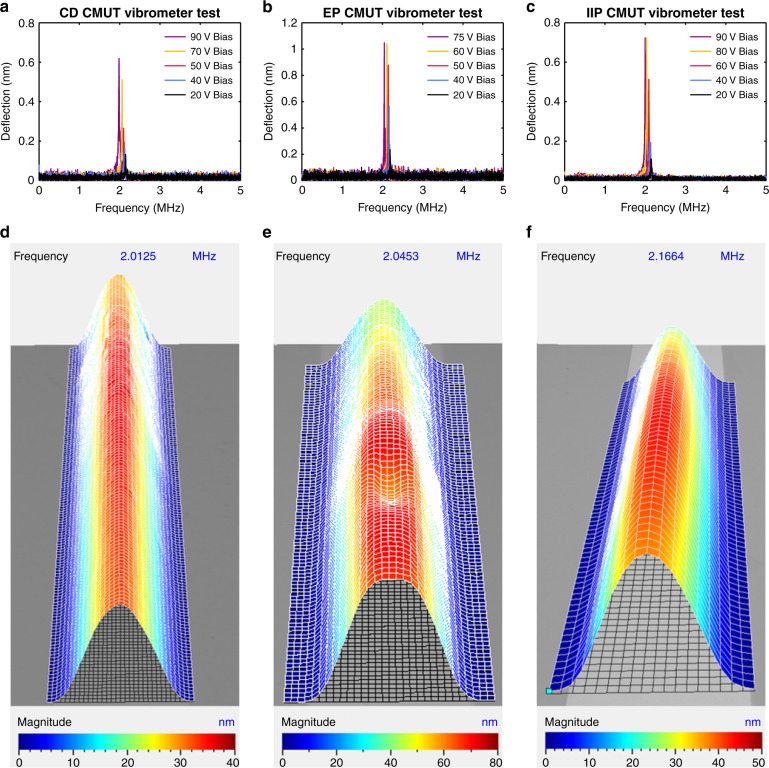
Laser Doppler vibrometry measurements. **a**–**c** Rectangular CMUT membrane deflection in response to 10 V pseudorandom signals applied at pre-collapse bias voltages. **d**–**f** Vibrometry scans of each rectangular CMUT architecture driven at resonance with a 10 V sinusoid. The CD, EP, and IIP rectangular CMUTs were biased at 70 V, 75 V, and 65 V, respectively

The dynamic operation of all architectures of long rectangular CMUTs was characterized in air using a laser Doppler vibrometer (MSA-500, Polytec, Baden-Württemberg, Germany). In these experiments, a single CMUT element was actuated at a time, with a bias tee (ZFBT-4R2GW-FT+, Mini-Circuits, Brooklyn, NY) used to superimpose an AC signal from the vibrometer onto an externally applied DC bias. To characterize the resonance frequency in air, the membrane deflection was measured while a pseudorandom signal with 10 V amplitude was applied. Fig. 5a–c illustrates the resonance frequency in air of each rectangular CMUT architecture at different pre-collapse bias voltages.

Once the resonance frequency was determined, the behavior of each membrane when operating at its resonance was examined. A sinusoid with a 10 V amplitude was applied to each device to drive it at its resonance frequency while the vibrometer scanned across the membrane measuring the displacement over time. A scan of each rectangular CMUT architecture, biased below the collapse voltage and driven at its resonance frequency, is displayed in Fig. 5d–f. Note that the field of view of the vibrometer was not sufficient to view the entire membrane, so the scan was performed at the center of the membrane.

For each architecture, the rectangular membrane oscillated in phase, with the maximum deflection near the center, defined as the (1,1) mode in^32^. At these pre-collapse voltages, there did not appear to be longitudinal modes or other complicated membrane behaviors.

### Impulse response

**Fig. 6:**
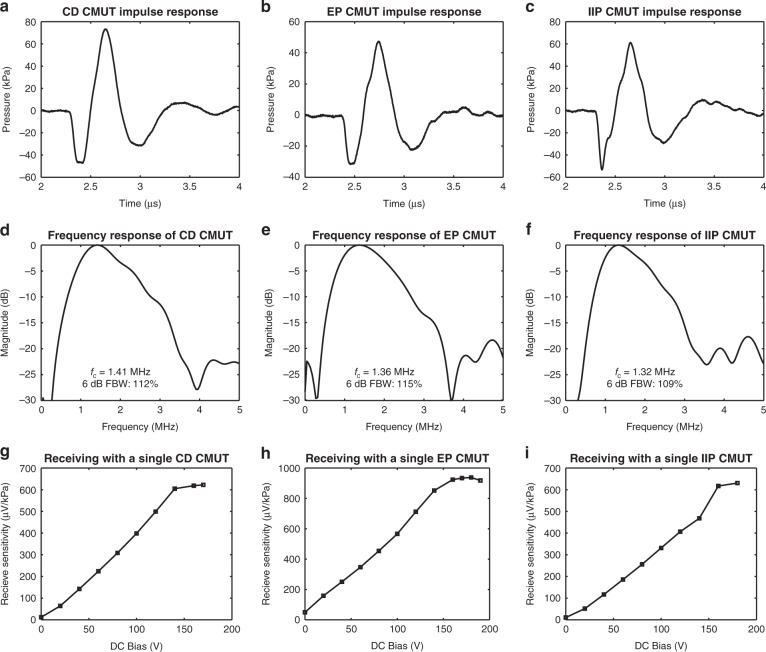
Acoustic testing. **a**–**c** Pressure signal emitted by each rectangular CMUT architecture in response to a 50 µJ impulse signal. **d**–**f** Frequency response of each rectangular CMUT architecture, as calculated from **a**–**c**. **g**–**i** Receive sensitivity measurements of each architecture of single-membrane CMUTs

To perform acoustic tests, small 14-element arrays of completed CMUTs were wire bonded to a custom printed circuit board (PCB) with an attached acrylic tank to hold oil. The impulse response of each CMUT architecture was determined by driving a single, unbiased CMUT element with a pulser receiver (5800PR, Olympus Corporation, Shinjuku, Tokyo, Japan) configured to output a very sharp negative voltage spike. The emitted pressure wave was detected using a needle hydrophone (HNP-400, Onda Corporation, Sunnyvale, CA) with a preamplifier (AH-2010-100, Onda Corporation). For each fabricated CMUT architecture, the pressure signal detected by a hydrophone placed 3.6 mm away from a single CMUT pulsed with 50 µJ of energy is given in Fig. 6a–c. Using the detected pressure signals, the fast Fourier transform (FFT) was calculated to characterize the frequency response of each element in oil (Fig. 6d–f).

### Receive sensitivity

The receive sensitivity of the fabricated CMUTs was investigated by transmitting ultrasound with a calibrated transducer and receiving with a single CMUT element. Based on the frequency response of the CMUTs, a suitable single-element piezoelectric transducer (XDR 058, Sonic Concepts, Bothell, WA) with a center frequency of 1.55 MHz was selected and calibrated using a needle hydrophone. Receive experiments were performed by placing the calibrated transducer 12.0 mm away from the CMUTs and applying 12.5 µJ pulses to the transducer using a pulser receiver. Using a bias tee, the DC voltage applied to the CMUT element was incremented, while the detected AC output was amplified externally by 29 dB. Based on these tests, the receive sensitivity as a function of bias voltage was calculated for each CMUT architecture, as displayed in Fig. 6g–i.

At maximum, a receive sensitivity of 623 µV/kPa from the CD device was observed, compared to 938 µV/kPa from the EP CMUT and 631 µV/kPa from the IIP device. The detected signals were also used to determine the noise equivalent pressure (NEP) of each CMUT architecture. The CD rectangular CMUT demonstrated a minimum NEP of 30.7 mPaHz^−1/2^, while the EP and IIP devices exhibited minimum NEP values of 21.5 mPaHz^−1/2^ and 40.3 mPaHz^−1/2^, respectively.

### Transmit sensitivity

The transmit sensitivity of each rectangular CMUT architecture was characterized by transmitting from a single CMUT element and detecting the signal using a hydrophone. A bias tee was used to superimpose an AC transmit signal from a function generator (AFG 3021B, Tektronix, Beaverton, OR) onto a DC bias from a power supply. The function generator was configured to output a 10 V_pp_ burst of 5 sinusoid pulses (assuming a matched 50 ohm load); however, the voltage across the CMUT was measured to be 20 V_pp_, as the impedance of our test setup was much higher. For each device, the driving sinusoid was set to 1.5 MHz because this frequency yielded the greatest output pressure for pre-collapse operation during preliminary tests. The hydrophone was aligned for maximum pressure 1 mm away from the CMUT. During the experiment, the CMUT bias was incremented from zero to beyond the collapse voltage, with the detected pressure waveform recorded at each voltage.

**Fig. 7:**
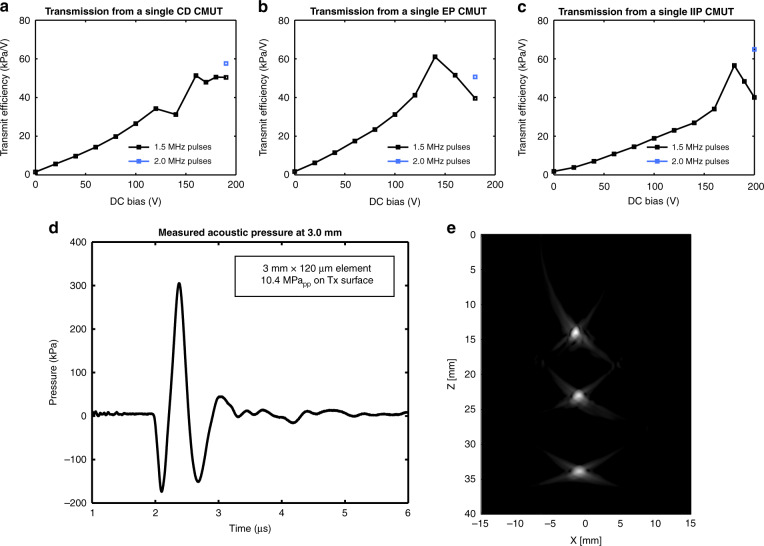
Transmit testing and imaging. **a**–**c** Transmit pressure on the surface of a CMUT membrane generated per AC volt as a function of bias voltage for CD, EP, and IIP rectangular CMUTs. **d** Acoustic pressure detected by a hydrophone 3.0 mm away from a contiguous dielectric single-membrane rectangular CMUT. **e** Effective flash image of three point-targets obtained by translating a single rectangular CMUT. This image is displayed with 40 dB log compression

Similar to other works^5,19,23^, we performed calculations accounting for diffraction and attenuation to determine the pressure on the transducer surface. Additional information regarding these calculations is available in Suppl. 3^5,19,21,23,33–35^. For each rectangular CMUT architecture, the surface pressure transmitted per AC volt applied (transmit efficiency) is given as a function of bias voltage in Fig. 7a–c. The maximum transmit efficiency was 51 kPa/V for CD CMUTs, 61 kPa/V for EP CMUTs, and 57 kPa/V for IIP CMUTs, with each device type achieving surface pressures greater than 1.1 MPa. During this test, there was clear evidence of charging in the CD devices at high bias voltages, leading to unreliable operation. In these regimes, the output amplitude and signal shape would often fluctuate, even during the oscilloscope averaging period. This effect was absent from tests on EP and IIP CMUTs, which were generally very stable.

As a comparison, we tested a commercial ultrasound probe (P4-2, Philips, Amsterdam, Netherlands) using a similar procedure. To characterize individual elements of the probe, the hydrophone was aligned for maximum pressure above each element using a custom water tank with a window for the probe. The hydrophone pressure was measured 2.3 mm from the transducer. Similar to the CMUT experiment, individual array elements were addressed with a burst of 20 V_pp_ sinusoids at the device center frequency (2.5 MHz) using a Verasonics Vantage system (Verasonics, Kirkland, WA). Accounting for diffraction and elevation focusing, the transmit efficiency of the piezoelectric transducer was determined to be 23.7 kPa/V.

To investigate device behavior at high, post-collapse voltages, additional tests were performed. Each CMUT architecture was biased at the maximum voltage from the previous tests, and the frequency of the transmit pulse train was varied to find the maximum output amplitude. All other parameters were kept constant. For each device, the maximum output pressure occurred when the function generator was set to produce 2.0 MHz sinusoidal pulses. With this driving signal, the CD, EP, and IIP CMUTs achieved surface pressures of 1.2 MPa, 1.0 MPa, and 1.3 MPa, respectively. As illustrated in Fig. 7a–c, this corresponds to transmit efficiencies of 58 kPa/V, 51 kPa/V, and 65 kPa/V using 2.0 MHz pulses for the CD, EP, and IIP CMUTs, respectively. Given that 1.5 MHz pulses optimized the output pressure at pre-collapse voltages, a shift in resonance frequency likely explains why the transmit efficiency decreased at high voltages in the initial experiment.

Additional transmit tests were performed using larger driving signals in order to investigate the maximum pressures achievable with these devices. Similar to previous experiments, these tests were performed by transmitting from a single CMUT and receiving with a hydrophone. The hydrophone was aligned for maximum pressure 3.0 mm away from the transmitting element. To drive the CMUT, a negative 170 V, 280 ns unipolar pulse was superimposed on a negative 60 V DC bias. At the location of the hydrophone, a 480 kPa_pp_ signal was detected from a single CD rectangular CMUT, as shown in Fig. 7d. This signal had a center frequency of 1.51 MHz, with a 6 dB fractional bandwidth of 93%. Accounting for diffraction and attenuation (0.15 dB/cm), this corresponds to a pressure of 10.4 MPa_pp_ on the transducer surface and a transmit efficiency of 61.2 kPa/V. These results are compared to surface pressure and transmit efficiency estimates reported in other works for piezoelectric materials and other CMUT architectures in Table 1.

**Table 1:** Transmit performance compared to other works

	PZT ceramic (PZ21)^a^	Single crystal PMN-0.33PT^a^	Commercial PZT Probe^b^	CMUT with substrate-embedded springs^b^	Dual-Mode CMUT with spacers^c^	This work
Frequency (MHz)	1.85	1.85	2.00	1.85	3.1	1.51
Max. Pressure (MPa)	0.924 @ 60V_pp_	2.46 @ 90V_pp_	0.802 @ 60V_pp_	1.88 @ 90V_pp_	1.7 @ 90V_pp_	10.4 @ 170V_pp_
Transmit Efficiency (kPa/V)	15.4	27.3	13.4	21.5	18.9	61.2

^a^From simulation results reported by ref. ^23^

^b^Characterization performed in ref. ^23^

^c^Characterization performed in ref. ^19^

Note that tests with larger transmit signals or larger DC biases often caused breakdown events in the bond pad regions of fabricated devices. As a result, several devices (of each architecture type) broke down during these experiments and other similar transmit tests with large signals. However, surface pressures of at least 6.0 MPa_pp_ and measured hydrophone pressures of 300 kPa_pp_ were achieved with each fabricated rectangular CMUT architecture in similar tests.

Finally, to investigate the use of single-membrane CMUTs in larger arrays, we performed a preliminary test translating a single CMUT element to form a synthetic array. Using a scanning stage (MLS203, Thorlabs, Newton, NJ), the CMUT element was translated in 150 µm steps, corresponding to an effective linear array with 200 elements and 150 µm pitch. This was used to achieve an effective flash image of point targets at various depths using a single-sinusoid pulse (125 V_pp_, 1.5 MHz). The reconstructed image is displayed at 40 dB compression in Fig. 7e. More details regarding this experiment are provided in Suppl. 3.

## Discussion

This work investigates three main ideas: designing CMUTs with a single rectangular membrane as opposed to multiple smaller membranes, incorporating electrode-posts or isolated isolation posts into an insulated cavity, and delaying the thermal oxidation step until after patterning the cavity during fabrication. Single-membrane CMUTs were intended to achieve good transmit performance and electromechanical efficiency, potentially competing with piezoelectric technology. As demonstrated in Table 1, the transmit capabilities of our single-membrane CMUTs compare favorably, not only to other high-output CMUT architectures but also to commercial transducers and simulations of single-crystal piezoelectric materials such as PMN-PT. In the 1.5–2.0 MHz range, a single rectangular CMUT element achieved transmit efficiencies of up to 65 kPa/V, 2–3 times greater than other reported results. This finding was verified in experiment, where our CMUTs obtained over 2.7 times the transmit efficiency of a commercial probe element. This exceptional transmit sensitivity allowed surface pressures of up to 10.4 MPa_pp_ to be achieved. Although even greater transmit pressures could be achieved in the future with phased array focusing and further device optimization, this promising result demonstrates the potential of our single-membrane rectangular CMUTs for both pulse-echo imaging and ultrasound therapy applications.

The fabricated single-membrane rectangular CMUT architectures exhibited excellent electromechanical efficiency, with *k*_*T*_^2^ values as high as 0.953 achieved. This value is higher than reported values for CMUTs and for piezoelectric transducers, which are nearly ubiquitous in commercial ultrasound systems. Compared to piezoelectric transducers, several authors have reported *k*_33_ values as high as 0.94 in single crystal structures of PMN-PT (lead magnesium niobate-lead titanate) or PIN-PMN-PT(lead indium niobate-lead magnesium niobate-lead titanate)^36–38^, corresponding to a maximum possible electromechanical conversion efficiency of almost 90% for longitudinal vibrations. However, for transducer array elements, electromechanical coupling is much better characterized by *k*_*T*_, which is typically considerably lower than *k*_33_^39,40^. For single crystal structures of PMN-PT or PIN-PMN-PT, reported *k*_*T*_ values typically range from 0.5 to 0.62^37,38^. These materials have been used in 1–3 composites to achieve *k*_*T*_ values of 0.855^39,41^, achieving much better coupling than for more standard piezoelectric materials such as PZT;^40^ however, even this corresponds to a *k*_*T*_^2^ value of 0.73, which is less efficient than the values achieved with our single-membrane CMUT architectures.

In CMUTs, typical designs achieve *k*_*T*_^2^ values of approximately 0.3 at 90% of the collapse voltage^4,18^, although specialized designs have achieved up to 0.82 at this point^5^. Although individual CMUT cells can achieve high electromechanical efficiency, the challenge with electromechanical efficiency in multiple-membrane CMUT designs is twofold. Not only does the multiple-membrane paradigm lead to suboptimal electromechanical efficiency, but the higher efficiency regimes close to the collapse voltage are often avoided due to charging and hysteresis. Our results suggest that single-membrane rectangular CMUTs are a viable solution to the former problem. To address the latter, we investigated the EP and IIP architectures.

Comparing the performance of the three fabricated architectures, both the EP and IIP devices mitigated hysteresis and showed negligible charging over more than 18 h of exposure to high electric fields and more than 500,000 collapse-snap-back cycles. The combined achievement of minimal charging and hysteresis-free operation enables reliable operation close to (or beyond) the collapse voltage where electromechanical efficiency is maximized.

IIP CMUTs exhibited slightly lower electromechanical efficiency and higher collapse voltages than typical contiguous dielectric designs. By contrast, EP CMUTs consistently exhibited higher electromechanical efficiency (and typically lower snap-down voltages) than their CD counterparts. As explored in Suppl. 1, this can be explained in terms of the electric field strength inside the cavity. In IIP CMUTs, the bottom electrode is patterned with electrically floating post regions, reducing the net electrostatic force on the membrane. In EP devices, the electrode is raised in the post regions. This decreased distance between electrodes leads to increased electrostatic attraction, with increasing impact on performance as the membrane deflects.

Remarkably, neither EP nor IIP structures appeared to alter the device operating frequency or considerably reduce the transmit-receive performance. In fact, the fabricated EP CMUTs consistently outperformed CD CMUTs in terms of receive sensitivity, generating AC signals with up to 50% greater amplitude under identical test conditions. This differs from the findings of Huang et al., who found that their PostCMUT architecture achieved 37.5% of the transmit sensitivity and 33% of the receive sensitivity of conventional contiguous dielectric CMUTs^17^. This discrepancy is likely explained by the fact that outside of the post regions, Huang’s PostCMUTs had a larger effective gap between electrodes than their conventional CMUT, leading to reduced performance. By contrast, all of our devices had the same separation between electrodes and contiguous insulation throughout the cavity, leading to identical effective gaps outside the post regions.

It is important to note that the long rectangular membranes in our devices generally did not exhibit complicated modes or additional unwanted resonances. Similar to the results reported in Zhang et al.^21^, our long membranes demonstrated a single resonance (and some higher frequency harmonics) in the MHz range when operated below the collapse voltage. The operating frequency of the elements was principally determined by the width and thickness of the membrane. At this resonance, the entire membrane oscillated in phase, with maximum deflection in the center (1,1 mode)^32^. Previously, Wong et al. investigated ensembles of smaller rectangular membranes and found that despite ideal (1,1) deflection modes occurring during air tests, dispersive higher order modes led to poor output pressure in immersion^32^. Based on our impulse response tests and very large output pressures, our single-membrane CMUTs did not appear to be susceptible to these effects.

It should be noted that collapse-mode operation in air was not fully investigated due to some limitations of our setup; thus, it is possible that more complicated behaviors may have occurred in this regime. This requires further investigation in future work. However, transmit and receive tests were performed well beyond the collapse voltage in immersion with good performance. In terms of noise equivalent pressure, fabricated rectangular CMUT designs achieved NEP values as low as 21.5 mPaHz^−1/2^. These results are comparable to CMUT designs in other works with similar dimensions^42^. Our results suggest that our single-membrane CMUT designs can be effectively operated in immersion for both pre-collapse and collapse modes to produce MHz ultrasound.

Finally, a fusion bonding process was demonstrated for fabricating insulated CMUT cavities with post structures and high bonding yield. This was achieved by delaying the thermal oxidation step until after patterning of the bottom electrode, avoiding both cusps from multiple oxidation steps and precharging of the oxide from large electric fields in RIE processes. Compared to traditional wafer-bonded fabrication, the primary drawback of this process is that the CMUT cavity height is determined by a silicon etch step as opposed to dry oxide growth. As the etch rate of silicon RIE processing is typically less consistent than the growth rate of thermal oxide, this may lead to coarser tolerances in the cavity height. It is likely that this drawback could be mitigated with further efforts to develop a consistent RIE recipe and more extensive chamber conditioning.

This paper focuses on optimizing the performance of individual CMUT elements, and future work will involve the fabrication and testing of imaging arrays. Finally, although the fabricated CMUT cavities were quite robust, the bond pads were susceptible to breakdown when very high voltages were applied. Future work could seek to improve robustness to high transmit and bias voltages with attention to the bond-pad areas. Wire bond encapsulation may help with this objective, and the use of high-K dielectrics should be explored.

## Conclusion

This work explores the viability of CMUT elements designed with a single long rectangular membrane, as opposed to an ensemble of smaller membranes within an element. These single-membrane CMUTs demonstrated very high electromechanical efficiency compared to typical multiple-membrane CMUTs as well as leading piezoceramics technology. In some cases, the fabricated rectangular CMUTs achieved electromechanical efficiencies of 0.95 or higher. During immersion measurements, these single-membrane CMUTs demonstrated noise equivalent pressure per unit area comparable to previously reported multiple-membrane devices but offered considerable improvements in transmit efficiency. Within the single-membrane paradigm, this work also compared the reliability and performance of three different CMUT architectures: typical contiguous dielectric (CD) CMUTs, electrode-post (EP) CMUTs, and isolated isolation post (IIP) CMUTs with additional insulation. Conventional CD devices were subject to known issues of charging and hysteresis, which made near-collapse and post-collapse operation impractical. The IIP devices exhibited slightly reduced electromechanical efficiency but demonstrated stable operation over long testing periods with no charging or hysteresis. EP CMUTs demonstrated the highest electromechanical efficiency and considerably more sensitive receive operation than the other architectures. Remarkably, the EP CMUTs also demonstrated comparable charging robustness to IIP CMUTs and minimal hysteresis, enabling reliable operation in high sensitivity regimes. All device types demonstrated excellent output pressure, outperforming a commercial piezoelectric probe in transmit efficiency by 2.4–2.7 times. Finally, a double-SOI fusion bonding process was proposed for the fabrication of these designs with a very high bonding yield. Given the high electromechanical efficiency, excellent reliability, and sensitive immersion performance of individual rectangular CMUT elements, our architectures offer considerable promise for future ultrasonic technologies.

## Supplementary information

Suppl. 1. Parallel Plate Analytical Modelling of Device Architectures

Suppl. 2. Fabrication Details

Suppl. 3. Additional Details About Transmit Experiments
