# Supplementary material for: Outperforming piezoelectric ultrasonics with high-reliability single-membrane CMUT array elements
Source: Microsyst Nanoeng. 2022 Jun 2;8:59. doi: 10.1038/s41378-022-00392-0 (PMC9162926; doi:10.1038/s41378-022-00392-0)
Supplement: Supplementary file 1 — Suppl. 1. Parallel Plate Analytical Modelling of Device Architectures [file 41378_2022_392_MOESM1_ESM.pdf]

Outperforming Piezoelectric Ultrasonics with High-Reliability Single-Membrane CMUT Array Elements

Eric B. Dew[1], Afshin Kashani Ilkhechi[1], Mohammad Maadi[1], Nathaniel J. M. Haven[1], and Roger J. Zemp[1,*]

[1]University of Alberta, Department of Electrical and Computer Engineering, Edmonton, T6G 1H9, Canada
[*]rzemp@ualberta.ca

Suppl. 1. Parallel Plate Analytical Modelling of Device Architectures

Even the simplest CMUT designs represent an extremely complicated system that is difficult to model analytically. However, a tractable analysis can be performed with some simplifying approximations. A first-order analysis useful for approximating the collapse voltage of contiguous dielectric (CD) CMUT designs was proposed by Ergun et al.[1] This simplified model was achieved by neglecting the curvature of the membrane (assuming piston-like motion), neglecting fringing electric fields, and modelling the mechanical restoring force as a linear spring constant.

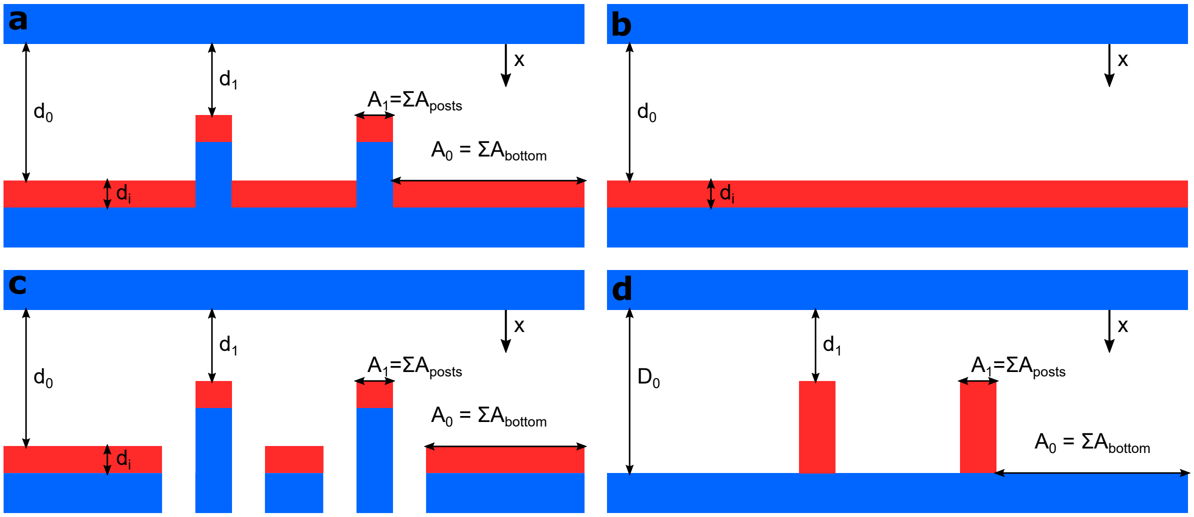

Figure 1. Simplified models of each CMUT architecture. **a** A model of an EP CMUT; **b** A model of a CD CMUT; **c** A model of an IIP CMUT; **d** A model of a PostCMUT as proposed by Huang et al.[2]

A similar analysis can be applied to electrode-post (EP) and isolated isolation post (IIP) CMUTs. As shown in Fig. 1a, an EP CMUT can be modelled as two parallel capacitors with separate gap distances. Making the same assumptions as in Ref. 1, the electrostatic force on the piston-like membrane can be expressed as

$$F_e = \frac{\varepsilon_0 A_0 V^2}{2(d_0 + \frac{d_i}{\varepsilon_r} - x)^2} + \frac{\varepsilon_0 A_1 V^2}{2(d_1 + \frac{d_i}{\varepsilon_r} - x)^2} \tag{1}$$

where F_e refers to the electrostatic force applied to the membrane; ε_0 is the permittivity of free space; V is the applied bias voltage; A_0 is the area of the CMUT cavity without posts; A_1 is the area of the isolation posts; d_0 is the height of the CMUT cavity; d_1 is the distance available for the membrane to deflect before contacting the isolation posts; d_i is the thickness of the insulating layer; ε_r is the relative permittivity of the insulating layer; and x is the membrane displacement. As in Ref. 1, the mechanical restoring force on the membrane is modelled as

$$F_m = kx \tag{2}$$

where F_m is the mechanical restoring force, and k is the spring constant. Equating the electrostatic force (1) and the mechanical restoring force (2), we obtain a relationship between the applied bias voltage and the membrane deflection

$$V = \sqrt{\frac{2kx}{\varepsilon_0}} \frac{(D_0 - x)(D_1 - x)}{\sqrt{A_0(D_1 - x)^2 + A_1(D_0 - x)^2}} \tag{3}$$

where $D_0 = d_0 + d_i/\varepsilon_r$, and $D_1 = d_1 + d_i/\varepsilon_r$. As with contiguous dielectric CMUTs, Equation (3) gives a physical solution up to the collapse-point where the restoring force cannot balance the electrostatic force. Following the method of Ref. 1, we differentiate (3) with respect to x, and set the derivative to zero in order to determine the membrane displacement at the point of collapse. This procedure yields the following quartic equation.

$$\begin{aligned} A_0D_0D_1^3 + A_1D_1D_0^3 - 3(A_0D_1^2D_0 + A_1D_0^2D_1 + A_0D_1^3 + A_1D_0^3)x \\ +3[A_0D_0D_1 + A_1D_0D_1 + 3(A_0D_1^2 + A_1D_0^2)]x^2 \\ -(9(A_0D_1 + A_1D_0) + A_0D_0 + A_1D_1)x^3 + 3(A_0 + A_1)x^4 = 0 \end{aligned} \tag{4}$$

The first root of (4) gives the membrane deflection at the collapse-point x_c, while the other three roots correspond to non-physical solutions. A general solution can be obtained for this root in terms of A_0, A_1, D_0, and D_1; however, it is omitted from this document as it is extremely long. In practice, it is easier to plot (3) and numerically determine the collapse-point, or substitute values of A_0, A_1, D_0, and D_1 into (4) and solve computationally. The physical solution to (4) can be substituted into (3) to determine the collapse voltage in terms of k and other design parameters.

In the case of a CD CMUT (Fig. 1b)without posts inside the cavity ($A_1 = 0$, $A_0 = A$), expressions for the electrostatic force (1) and voltage (3) reduce to those derived by Ergun et al. (Ref. 1, eq. (1-3)). In the case of an IIP CMUT, (Fig. 1c) the posts are electrically floating. Other works have demonstrated that it is possible to store charge in floating silicon by directly applying extremely high bias voltages to the floating electrode and then disconnecting it from the high voltage supply[3,4]. However, this differs from our design where a bias voltage is not applied to the floating posts, and thus it is unlikely that substantial charge is stored in these regions. Assuming that no charge is stored in the floating silicon, the electric field between the top and bottom electrode in these regions is minimal (neglecting fringing electric fields), and the second term of (1) vanishes. Thus, the electrostatic force in an IIP CMUT is described by (5).

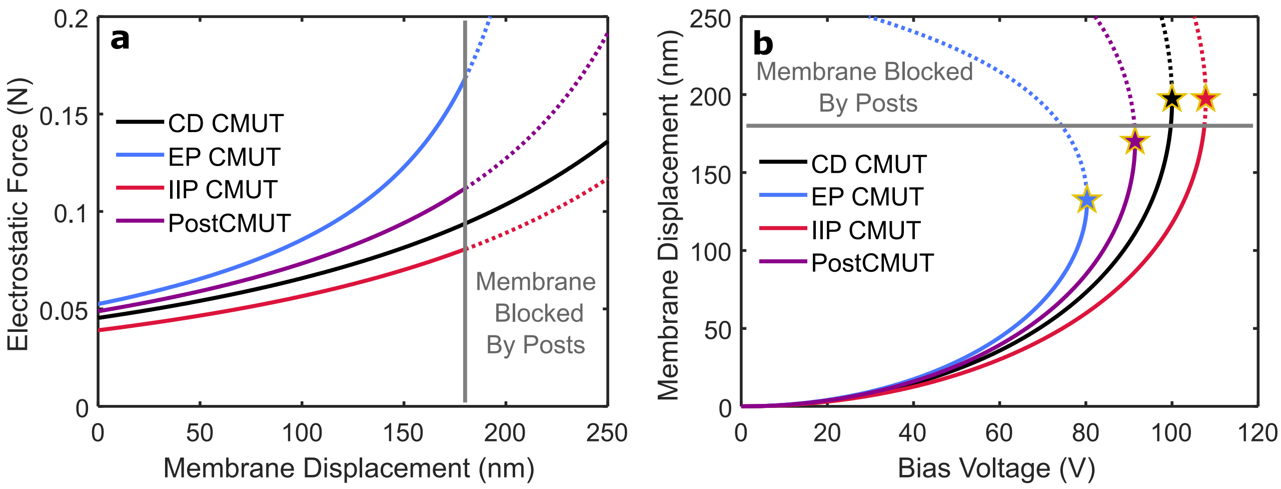

Figure 2. Fig. 2 **a** Electrostatic force as a function of membrane displacement calculated for each architecture using our simplified model, with the Bias voltage $V = 100\,\mathrm{V}$. The grey line indicates the region beyond which posts stop the membrane from deflecting in EP, IIP, and PostCMUT devices. **b** Membrane displacement calculated as a function of bias voltage for each architecture using our analytical model, with the collapse point indicated by a star. The spring constant k was taken to be $517\,\mathrm{kNm}^{-1}$ for this calculation. The grey line indicates the region beyond which posts stop the membrane from deflecting in EP and IIP devices. Dotted lines indicate a non-physical portion of the curve due to membrane collapse or posts preventing the membrane from deflecting further.

$$F_e = \frac{\varepsilon_0 A_0 V^2}{2(d_0 + \frac{d_i}{\varepsilon_r} - x)^2} \tag{5}$$

Note that (5) is nearly identical to the expression for a CD CMUT (Ref. 1, eq. (1)), the only difference being that the area without posts A_0 appears in (5) instead of the total CMUT cavity area A. Thus, the relationship between the electrostatic force in an IIP device and a CD CMUT is given by (6).

$$F_{e_{IIP}} = \frac{A_0}{A} F_{e_{CD}} \tag{6}$$

Notably, this means that the collapse-point occurs at the same deflection as a CD CMUT ($x_c = D_0/3$)[1] and the voltage required to achieve a given membrane deflection is given by (7).

$$V_{IIP} = \sqrt{\frac{2kx}{A_0 \varepsilon_0}}(D_0 - x) = \sqrt{\frac{A}{A_0}} V_{CD} \tag{7}$$

Equations (1) and (3) can also be modified to model the PostCMUT architecture presented in Huang et al.[2] As shown in Fig. 1d this architecture can also be modelled as two parallel capacitances, with slightly different effective gap distances between electrodes than modelled in (1). To assess the impact of the elevated electrodes in our EP architecture, we also modelled the PostCMUT architecture. This was performed using an equivalent effective gap D_0 between the electrodes outside of the post regions, and dielectric posts with equal height to the EP case.

The electrostatic force applied to the membrane for a given bias voltage is plotted as a function of membrane deflection for each modelled CMUT architecture in Fig. 2a using approximate dimensions from our fabricated devices. Although a given bias voltage will only correspond to one point on this curve at steady state, the membrane deflection may be altered by impinging pressure waves or oscillations during operation. Thus, the total electrostatic force on the membrane can provide some insight into device sensitivity. Notably, the EP architecture has advantages over other architectures due to the reduced effective gap between electrodes in the post regions, while the IIP architecture has reduced sensitivity due to the patterned bottom electrode.

The modelled membrane deflection is also plotted as a function of bias voltage for each architecture in Fig. 2b, providing insight into the membrane collapse voltage and hysteresis. The calculations were performed using $A = 3.7 \times 10^{-7}\,\text{m}^2$; $d_i = 360\,\text{nm}$; $\varepsilon_r = 3.9$; $d_0 = 500\,\text{nm}$; $d_1 = 180\,\text{nm}$. For EP CMUTs and PostCMUTs, area fractions of $A_0 = 0.958A$ and $A_1 = 0.042A$ were used. For IIP CMUTs $A_0 = 0.861A$ was used.

Based on this model, CD, EP, and IIP devices are predicted to collapse at 100.0 V, 80.3 V, and 107.8 V respectively. These collapse voltage predictions are compared to experimental collapse voltage results in Table 1. For CD and EP CMUTs, the predicted collapse voltage is well within 1 standard deviation of the experimental mean collapse voltage, indicating reasonable agreement between theory and experiment given the number of simplifying approximations used. Our model prediction was within 1.2 standard deviations of the mean collapse voltage for IIP CMUTs, indicating slightly worse agreement between theory and experiment. It is likely that much of this discrepancy arises from neglecting fringing electric fields and membrane curvature in our calculation. In our model, all active bottom electrode area is valued equally due to the piston-like-motion approximation. In reality, the area near the center of the cavity where the membrane is deflected the most will contribute more electrostatic force than areas near the edge. However, these high-value areas of the electrode are also the locations where IIPs (which do not contribute substantial electrostatic force) are placed to prevent membrane collapse. It is therefore unsurprising that our model under-estimates the difference in collapse voltage between CD and IIP devices.

CMUT Architecture	Modelled Collapse Voltage (V)	Experiment Mean Collapse Voltage (V)	Collapse Voltage Standard Deviation (V)
CD	100.0	93.6	9.0
EP	80.3	84.1	4.5
IIP	107.8	118.3	8.8

Table 1. Comparison of Model Predictions and Experimental Collapse Voltages

To investigate hysteresis, the deflection curve was derived for each CMUT architecture based on Equation (3) and Fig. 2b. The snap-back voltage was derived by equating the electrostatic force (1, 5) with the mechanical restoring force (2) at the collapsed distance (d_1 for devices with posts, d_0 for a CD CMUT) and solving for V. These calculated deflection curves are shown in Fig. 3. Note that this model predicts a greater amount of hysteresis than observed in experiment for both CD and EP CMUTs. This discrepancy likely arises from neglecting the membrane curvature in our calculations. In reality, the

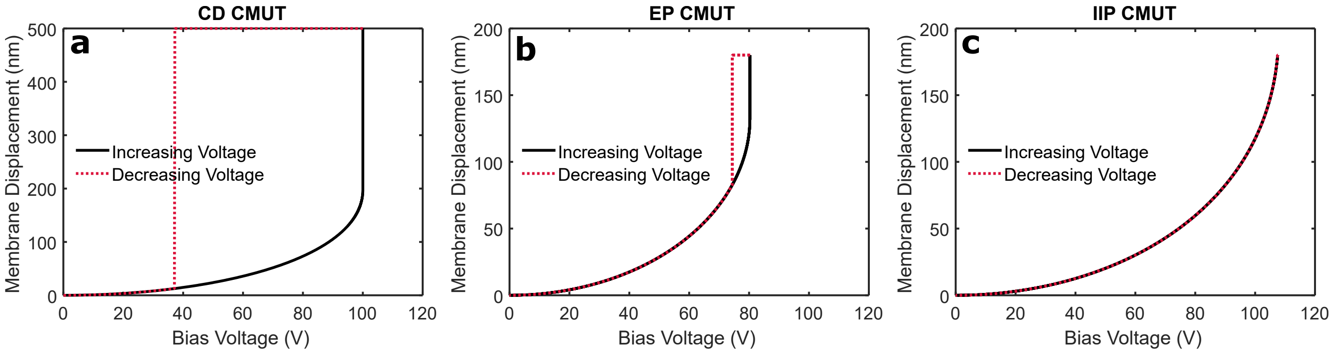

Figure 3. Predicted membrane deflection as a function of bias voltage for each CMUT architecture as calculated from our parallel plate model. **a** Contiguous dielectric (CD) CMUT with no posts. **b** Modified isolation post (IP) CMUT. **c** Isolated isolation post (IIP) CMUT.

average deflection of the membrane is much less than the maximum deflection. This is particularly important when considering the impact of EPs, as the electrostatic force that the post regions contribute is much greater when the membrane is deflected further. Essentially, assuming piston-like motion over-estimates the impact of the electrode-post regions on the collapse voltage. Given this consideration, it is unsurprising that the difference between EP and CD CMUT collapse voltage was smaller in experiment than in our model. This also explains why our model predicted substantially more hysteresis than what we observed in experiment for EP devices. However, it should be noted that as the electrostatic force is increased in post regions for both EP CMUTs and PostCMUTs, the height cannot be perfectly tuned to completely negate hysteresis (although it can be minimized to the point where it is negligible). By contrast, in IIP CMUTs the post height does not impact the collapse voltage (as there is ideally no electrostatic force in these regions). Thus, it is possible to design IIPs to completely negate hysteresis, as occurs in our design.

Finally, a sensitivity analysis was conducted to gain further insights into the device performance of each CMUT architecture. This was achieved by differentiating the capacitance of each device with respect to x, with the EP and PostCMUT architectures modelled as two parallel capacitances as in the previous calculations. The sensitivity of each device type to changes in membrane deflection $\frac{dC}{dx}$ is depicted in Fig. 4. As expected, the EP devices are the most sensitive due to the heightened electric field in the post regions, whereas the IIP CMUTs are the least sensitive due to the lack of electrostatic attraction in the floating post regions. Note that this curve closely resembles the electrostatic force plot in Fig. 2a, as these quantities are related by a factor of $\frac{V^2}{2}$.

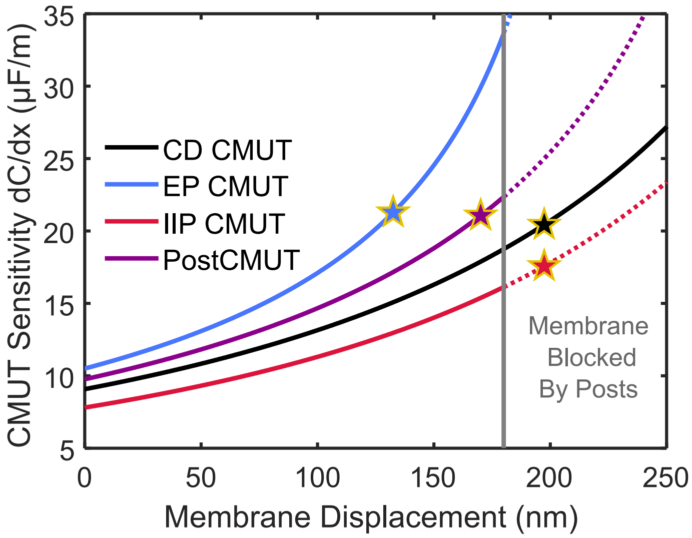

Figure 4. Calculated CMUT sensitivity to membrane displacement for each architecture. Dotted lines indicate a non-physical portion of the curve due to posts precluding further membrane deflection. Star-shaped markers indicate the collapse point assuming each device is biased at the collapse voltage.

References

1. Ergun, A. S., Yaralioglu, G. G. & Khuri-Yakub, B. T. Capacitive micromachined ultrasonic transducers: Theory and technology. *J. aerospace engineering* **16**, 76–84 (2003).

2. Huang, Y. *et al.* Capacitive micromachined ultrasonic transducers (CMUTs) with isolation posts. *Ultrasonics* **48**, 74–81 (2008).

3. Ho, M.-C., Kupnik, M., Vaithilingam, S. & Khuri-Yakub, B. T. Fabrication and model validation for CMUTs operated in permanent contact mode. In *2011 IEEE International Ultrasonics Symposium*, 1016–1019 (IEEE, 2011).

4. Ho, M.-C., Kupnik, M., Park, K. K. & Khuri-Yakub, B. T. Long-term measurement results of pre-charged CMUTs with zero external bias operation. In *2012 IEEE International Ultrasonics Symposium*, 89–92 (IEEE, 2012).
