# Supplementary material for: Outperforming piezoelectric ultrasonics with high-reliability single-membrane CMUT array elements
Source: Microsyst Nanoeng. 2022 Jun 2;8:59. doi: 10.1038/s41378-022-00392-0 (PMC9162926; doi:10.1038/s41378-022-00392-0)
Supplement: Supplementary file 2 — Suppl. 2. Fabrication Details [file 41378_2022_392_MOESM2_ESM.pdf]

Outperforming Piezoelectric Ultrasonics with High-Reliability Single-Membrane CMUT Array Elements

Eric B. Dew[1], Afshin Kashani Ilkhechi[1], Mohammad Maadi[1], Nathaniel J. M. Haven[1], and Roger J. Zemp[1,*]

[1]University of Alberta, Department of Electrical and Computer Engineering, Edmonton, T6G 1H9, Canada
[*]rzemp@ualberta.ca

Suppl. 2. Fabrication Details

Fig. 1 summarizes the difference between our approach and previous approaches to fusion bonding processes for CMUT fabrication. The fusion bonding process used in this work is summarized in Fig. 2, demonstrating fabrication of an isolated isolation post (IIP) single-membrane CMUT. Differences in the first three patterning steps between contiguous dielectric (CD), electrode-post (EP), and IIP CMUTs are explained in Fig. 3. After the third mask step, the remaining fabrication steps are identical for each architecture, allowing each device type to be fabricated on the same wafer. The remaining portions of this document describe fabrication steps in detail, and discuss previous iterations of this process.

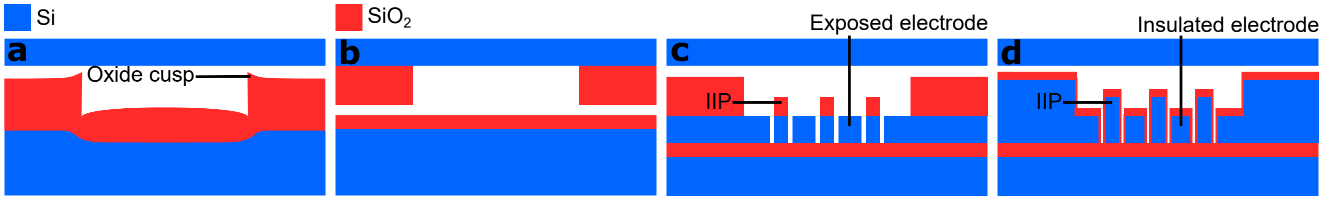

Figure 1. Cross sections of CMUT cavities during the fusion bonding step of different fabrication strategies. **a** Process with multiple oxidation steps (double oxidation process). **b** Oxide-oxide bonding process proposed by Christiansen et. al.[1] **c** IIP fabrication process proposed by Greenlay[2]. **d** Our proposed process, delaying thermal oxidation until after cavity structures are patterned into silicon.

Patterning Bottom Substrate

Processing begins on the bottom SOI wafer (Fig. 2a). The first step performed is a standard piranha cleaning process, in which the wafer is submerged in a 3:1 mixture of sulphuric acid (96%) and hydrogen peroxide (30%) for 15 minutes. This step is used to remove organic contaminants and prepare the wafer for HMDS (hexamethyldisilazane) treatment. Immediately following the piranha cleaning (and subsequent rinsing steps), the wafer is treated with HMDS and the first lithography step is performed. Piranha cleaning and subsequent HMDS treatment is performed before every lithography step except the final lithography to improve resist adhesion and minimize lithographic defects. The first lithography step defines the post structures in EP and IIP CMUTs as shown in Fig. 2b while in contiguous dielectric elements the entire cavity is left exposed (Fig. 3). In all device architectures, this lithography step begins to define the trench isolating each element from parasitic capacitance, although the trench is not etched to completion in this mask step. A standard 1.2 µm thick positive resist (HPR 504) is used for all lithography steps. Following the lithography step, the exposed silicon is then etched by approximately 320 nm using a Cl_2-based reactive ion etching (RIE) recipe (Oxford PlasmaPro 100 Cobra). The second lithography step defines the CMUT cavity. The mask is the same for each CMUT architecture in this step, with the entire cavity exposed as illustrated in Fig. 2c. Strictly for fabricating CD rectangular CMUTs, this step could be skipped and the first etch could be performed for longer as the masks are the same, however for EP and IIP structures this step determines how much room is available for the membrane to deflect before touching the posts. Following lithography, the exposed silicon is etched using the same Cl_2-based RIE process as the first etch, this time for approximately 180 nm. The third mask step is used to define the trenches around the isolated isolation post structures as depicted in Fig. 2d. In the EP and CD architectures, the entire cavity is protected with photoresist for this step, but the trench surrounding the element is exposed. This patterning step uses a Bosch deep reactive ion etching (DRIE) process to etch through the device layer with a high aspect ratio (Oxford PlasmaPro 100 Estrelas).

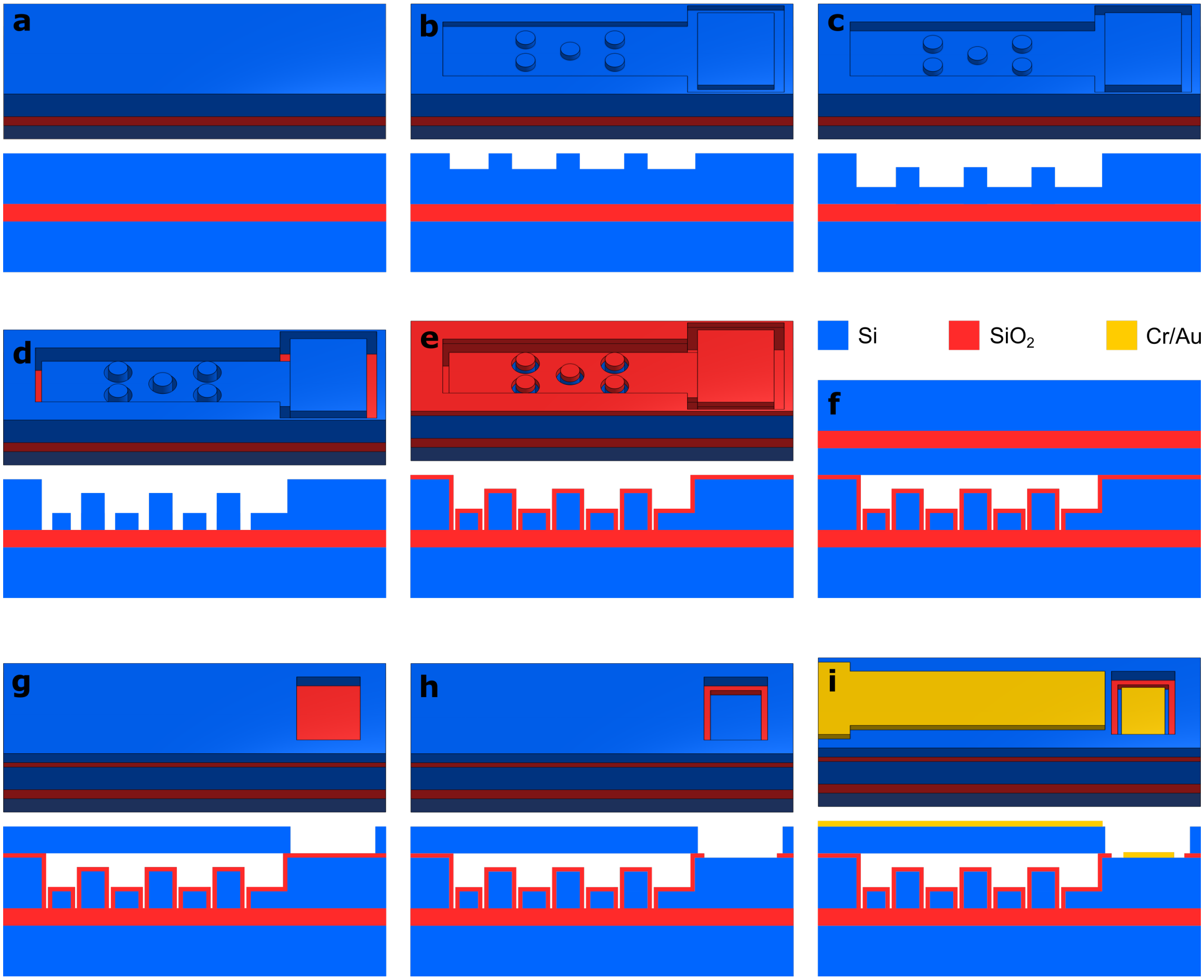

Figure 2. Simplified 3D drawing (above) and cross-sectional view (below) of a rectangular IIP CMUT during each step of fabrication **a** Bottom SOI wafer before processing. **b** First lithography and etching, defining posts from the bottom electrode. **c** Second patterning step etching down the entire cavity. **d** Third mask step and DRIE, etching through the device layer, isolating IIP structures and completing trenches around elements. **e** Thermal oxidation of bottom SOI wafer. **f** Fusion bonding and annealing of top and bottom SOI wafers. **g** Handle removal and fourth lithography step, etching through the membrane to access bottom electrode. **h** Fifth mask step, etching through thermal oxide to access bottom electrode. **i** Contact metal deposition and patterning.

Bonding Preparation

Following the DRIE etching of the device layer, a dry oxidation step is performed on the bottom wafer as illustrated in Fig. 2e. This oxidation step occurs at 1100°C, growing a 360 nm SiO_2 layer with good uniformity and no cusps or protrusions. Immediately prior to thermal oxidation, the wafer is thoroughly cleaned with piranha solution as described above to minimize oxide contaminants. Following oxidation of the bottom SOI wafer, the top SOI and processed bottom wafer are prepared for fusion bonding with an RCA clean step. All steps in the RCA clean process are performed including the oxide strip, which has little impact on the thermal oxide due to the low concentration of HF and low exposure time. Immediately following the completion of the cleaning process, the top and bottom wafers are fusion bonded in 5 mTorr vacuum (SUSS MicroTec CB6L) as depicted in Fig. 2f. For batch processes of wafers, the wafers awaiting bonding remain in high purity DI water inside the RCA rinse containers to limit exposure to airborne contaminants. The bonded wafer pair is then annealed at 1100°C to form covalent bonds, completing the bonding process.

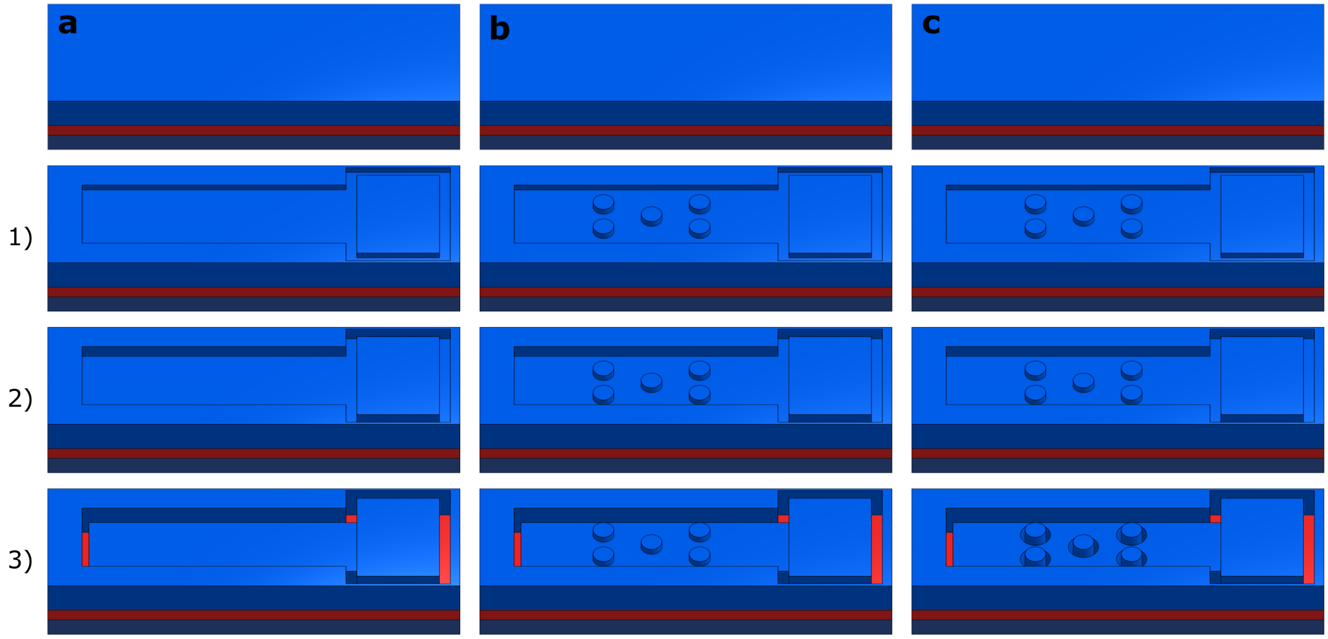

Figure 3. First three patterning steps for each fabricated CMUT architecture. **a** Contiguous dielectric (CD) rectangular CMUT, **b** Electrode post (EP) rectangular CMUT, **c** Isolated isolation post (IIP) rectangular CMUT. After the third mask step, all fabrication steps are the same for each architecture.

Membrane Release

Before bulk etching of the top SOI handle to release the membrane, PECVD oxide is deposited on the bottom wafer for protection. Bulk etching of the top wafer's handle is achieved using tetramethylammonium hydroxide (TMAH). A secondary benefit of performing the oxidation step much later in this process compared with typical wafer-bonded processes is that no etching steps have been performed on the thermal oxide layer. As such, the entire bottom wafer has a protective layer of high-quality thermal oxide underneath the PECVD oxide layer. This helps mitigate unwanted etching of the bottom wafer during TMAH processes, which may occur near imperfections in the PECVD oxide layer. As a result, long TMAH etches were less damaging to the bottom wafer and there were fewer issues with wafer fragility in later process steps compared to the process demonstrated by Greenlay[2]. However, etching most of the handle with DRIE prior to TMAH etching can further minimize these issues. After handle removal, the BOX layer is removed using buffered oxide etch (BOE).

Accessing Bottom Electrode

The fourth lithography step (Fig. 2g) defines access holes in the silicon membrane, which are etched using DRIE. The masks are designed such that the access holes from lithography 4 fall inside the boundary of the isolating trench, thus the hermetic seal inside the CMUT cavity and trench is not breached by the access hole (15.5 µm alignment tolerance). Fig. 2h illustrates the fifth patterning step, which etches access holes in the thermal oxide (RIE, CHF_3 and O_2) exposing the bottom electrode. The access hole etched into the oxide is smaller than that etched into the silicon, leaving an oxide rim that limits the potential for breakdown through the bond pad. Prior to contact deposition, the native oxide is removed and a piranha clean is performed. Metal contacts (Al or Cr/Au) are deposited using magnetron sputtering to a thickness of 250 nm. Finally, the sixth lithography step is performed, defining the bond pads and top electrode as illustrated in Fig. 2i. This lithography requires a high exposure dose as it is difficult to control the thickness of photoresist in the access holes. Metal contacts are wet etched as sputter etching risks redeposition inside the access holes.

Previous Process Iterations

Our process was repeated three times, with several 100 mm wafers per iteration. Each fabrication run had high bonding yield. However, device reliability and performance varied depending on the starting wafers used. Our third and final iteration generation of rectangular CMUTs was used for all experiments discussed in the main manuscript.

The first generation of rectangular CMUT devices was fabricated with using identical silicon-on-insulator (SOI) wafers from Ultrasil (Hayward, CA, USA) for the top and bottom substrate. The wafers featured 5 µm thick device-layers boron-doped to 0.01-0.02 Ωcm resistivity, a 500 nm buried oxide layer (BOX), and a 625 µm handle. The IIP CMUTs from this fabrication

were free of hysteresis and displayed excellent robustness to charging, with one device showing no observable CV-shifts over 1 million snap-down events and 48 hours of testing. However, these devices had poor performance in immersion, likely due to the relatively high resistivity in the bottom electrode[3].

The second generation of rectangular CMUTs also used two identical 100 mm SOI wafers from Ultrasil. In this case, the wafers had a 5 µm thick device layer with 0.001-0.005 Ωcm resistivity (boron doped), a 400 µm handle, and a 0.5 µm BOX layer. These CMUTs had much improve immersion performance, however the oxide quality was poor. As a result, the devices were susceptible to charging even at pre-collapse voltages, although fabricated EP and IIP architectures were still more robust than CD CMUTs. The poor oxide quality in these devices was not well understood but may have been due to the starting material. Future work is needed to better understand this.

The final iteration of this process used leftover wafers from the second generation for the top SOI (determining membrane properties) but used a different set of wafers purchased from Silicon Valley Microelectronics (Santa Clara, CA, USA) for the bottom electrode. The bottom substrates were specified to have a 10 µm device-layer with <0.005Ωcm device-resistivity (arsenic doped), 300 nm BOX thickness, and a 500 µm handle.

The final generation of rectangular CMUTs combined the best attributes of the two previous attempts. As the bottom electrode was heavily doped with arsenic, resistivity was low and all fabricated device types had good performance in immersion. Despite similarly high doping levels, the thermal oxide quality was also acceptable. It is possible that this difference is explained by differences in dopant behaviour, as arsenic has been found to diffuse far less into oxide at Si-SiO_2 interfaces[4] and leads to fewer interface states in thermal oxide grown on highly doped silicon[5].

References

1. Christiansen, T. L. *et al.* Void-free direct bonding of CMUT arrays with single crystalline plates and pull-in insulation. In *2013 IEEE International Ultrasonics Symposium (IUS)*, 1737–1740 (IEEE, 2013).

2. Greenlay, B. A. *Fabrication and Integration of CMUT Sensors*. Master's thesis, University of Alberta (2017).

3. Havreland, A. S. *et al.* CMUT electrode resistance design: Modeling and experimental verification by a row-column array. *IEEE transactions on ultrasonics, ferroelectrics, frequency control* **66**, 1110–1118 (2019).

4. Grove, A., Leistiko Jr, O. & Sah, C.-T. Redistribution of acceptor and donor impurities during thermal oxidation of silicon. *J. Appl. Phys.* **35**, 2695–2701 (1964).

5. Snel, J. The doped Si/SiO2 interface. *Solid-State Electron.* **24**, 135–139 (1981).
