# Supplementary material for: Outperforming piezoelectric ultrasonics with high-reliability single-membrane CMUT array elements
Source: Microsyst Nanoeng. 2022 Jun 2;8:59. doi: 10.1038/s41378-022-00392-0 (PMC9162926; doi:10.1038/s41378-022-00392-0)
Supplement: Supplementary file 3 — Suppl. 3. Additional Details About Transmit Experiments [file 41378_2022_392_MOESM3_ESM.pdf]

Outperforming Piezoelectric Ultrasonics with High-Reliability Single-Membrane CMUT Array Elements

Eric B. Dew[1], Afshin Kashani Ilkhechi[1], Mohammad Maadi[1], Nathaniel J. M. Haven[1], and Roger J. Zemp[1,*]

[1]University of Alberta, Department of Electrical and Computer Engineering, Edmonton, T6G 1H9, Canada
[*]rzemp@ualberta.ca

Suppl. 3. Additional Details About Transmit Experiments

Transmit experiments were performed by transmitting from a single CMUT element and receiving on a needle hydrophone a known distance away. When reporting transmit pressures, it is common to perform calculations accounting for diffraction and attenuation, thus determining the pressure on the transducer surface[1–3]. To calculate the pressure at the surface of our devices, we used an effective back-propagation approach. First, we used ANSYS to calculate the deflection profile for our rectangular membranes, as previously described in Zhang et al.[4] The normalized deflection profile was then used as an effective apodization for angular spectrum method simulations, which estimated the pressure profile in the observation plane of the hydrophone.[5] As the hydrophone was aligned for maximum pressure, the ratio of the maximum pressure in this observation plane to the modelled source pressure was used as a correction factor applied to the hydrophone data to then estimate the pressure at the surface. To account for attenuation propagating in vegetable oil, we used an attenuation coefficient of 0.15 dB/cm for 1.5 MHz ultrasound, and 0.27 dB/cm for 2.0 MHz ultrasound.[6,7]

Table 1 shows the measured hydrophone pressure for each device type, and the corrected pressure on the transducer surface. Note that other devices of comparable dimensions have had similar ratios between the measured hydrophone pressure and corrected surface pressure[3]. The distance used in our calculations was determined based on the time of flight, measured as the time between the start of the transmit pulse and start of the detected hydrophone signal. As this signal was sampled at 1 GS/s, this provides an accurate measure of the signal propagation distance. Note that our setup had to be reassembled between the 1.5 MHz and 2.0 MHz measurements on the IIP CMUT, hence there was a small discrepancy in the hydrophone position. This was accounted for in our calculations. Finally, note that the commercial PZT transducer element had an active area of 10.8 times greater than that of the CMUT elements. Thus, much less of the surface pressure was lost to diffraction compared to the CMUT measurements.

Device Type	Frequency (MHz)	Distance (mm)	Maximum Detected Hydrophone Pressure (kPa)	Calculated Surface Pressure (MPa)	Transmit Efficiency (kPa/V)
CD CMUT	1.5	0.90	79	1.1	51
CD CMUT	2.0	0.90	98	1.2	58
EP CMUT	1.5	1.15	80	1.3	61
EP CMUT	2.0	1.15	79	1.0	51
IIP CMUT	1.5	1.03	80	1.2	57
IIP CMUT	2.0	0.95	107	1.3	65
Commercial PZT Transducer	2.5	2.28	142	0.5	24

Table 1. Surface Pressure Calculated From Measured Hydrophone Pressure.

One challenge during our measurements was determining the voltage applied to the transmitting element on the commercial probe. This was accomplished by measuring the open load voltage from our Verasonics system, then accounting for the probe impedance to determine the voltage applied to the transducer element. Note that the open load voltage changed depending on the script being executed, so it had to be characterized for programs pertaining to our specific transducer. Once this was calibrated appropriately, the voltage applied to the probe was set to be 20 V_{pp}, (corresponding to an open-circuit voltage of approximately 40 V_{pp}) with a similar driving waveform to the CMUT tests. Also note that the elevational focusing lens was

accounted for in our angular spectrum method simulation, although the impact was small.

Preliminary Imaging Experiment

To investigate the use of single-membrane CMUTs in larger arrays, we performed a preliminary test translating a single CMUT element to form a synthetic array. The CMUT element was translated with respect to a needle hydrophone using a scanning stage (Thorlabs MLS203) in 150 μm steps, corresponding to an effective linear array with 200 elements and 150 μm pitch. To achieve an effective flash image, a 125 V_{pp} pulse comprised of a single 1.5 MHz sinusoid was applied without electronic delay to an unbiased CMUT at each transmit position. While we are transmitting on a translated element and receiving with a hydrophone, by reciprocity this is equivalent to imaging a point source emitter with an effective linear array. These measurements were repeated at three different hydrophone depths, after which the images were summed together.

References

1. Guldiken, R. O., Zahorian, J., Yamaner, F. & Degertekin, F. L. Dual-electrode CMUT with non-uniform membranes for high electromechanical coupling coefficient and high bandwidth operation. *IEEE transactions on ultrasonics, ferroelectrics, frequency control* **56**, 1270–1276 (2009).

2. Mahmud, M. M. *et al.* An improved CMUT structure enabling release and collapse of the plate in the same Tx/Rx cycle for dual-frequency acoustic angiography. *IEEE Transactions on Ultrason. Ferroelectr. Freq. Control.* **67**, 2291–2302 (2020).

3. Lee, B. C., Nikoozadeh, A., Park, K. K. & Khuri-Yakub, B. T. High-efficiency output pressure performance using capacitive micromachined ultrasonic transducers with substrate-embedded springs. *Sensors* **18**, 2520 (2018).

4. Zhang, P., Fitzpatrick, G., Harrison, T., Moussa, W. A. & Zemp, R. J. Double-SOI wafer-bonded CMUTs with improved electrical safety and minimal roughness of dielectric and electrode surfaces. *J. microelectromechanical systems* **21**, 668–680 (2012).

5. Zemp, R. J., Tavakkoli, J. & Cobbold, R. S. Modeling of nonlinear ultrasound propagation in tissue from array transducers. *The J. Acoust. Soc. Am.* **113**, 139–152 (2003).

6. Chanamai, R. & McClements, D. J. Ultrasonic attenuation of edible oils. *J. Am. Oil Chem. Soc.* **75**, 1447–1448 (1998).

7. Oralkan, O. *et al.* Capacitive micromachined ultrasonic transducers: Next-generation arrays for acoustic imaging? *IEEE transactions on ultrasonics, ferroelectrics, frequency control* **49**, 1596–1610 (2002).
